# Maternal Supplementation with Oligofructose (10%) during Pregnancy and Lactation Leads to Increased Pro-Inflammatory Status of the 21-D-Old Offspring

**DOI:** 10.1371/journal.pone.0132038

**Published:** 2015-07-06

**Authors:** Laís Vales Mennitti, Lila Missae Oyama, Juliana Lopez de Oliveira, Ana Claudia Losinskas Hachul, Aline Boveto Santamarina, Aline Alves de Santana, Marcos Hiromu Okuda, Eliane Beraldi Ribeiro, Claudia Maria da Penha Oller do Nascimento, Luciana Pellegrini Pisani

**Affiliations:** 1 Programa de Pós Graduação Interdisciplinar em Ciências da Saúde, Universidade Federal de São Paulo, Santos, SP, Brazil; 2 Departamento de Biociências, Instituto de Saúde e Sociedade, Universidade Federal de São Paulo, Rua Silva Jardim, 136, Térreo, Vila Mathias, Santos, SP, Brazil; 3 Departamento Fisiologia, Disciplina de Fisiologia da Nutrição, Escola Paulista de Medicina, Universidade Federal de São Paulo, Rua Botucatu, 862, 2° andar, Vila Clementino, São Paulo, SP, Brazil; Monash University, AUSTRALIA

## Abstract

Previously, we showed that oligofructose (10%) supplementation during pregnancy and lactation increased endotoxemia in 21-d-old pups. The present study evaluated the effect of 10% oligofructose diet supplementation during pregnancy and lactation in the presence or absence of hydrogenated vegetable fat on the pro-inflammatory status of 21-d-old offspring. On the first day of pregnancy, female Wistar rats were divided into the following groups: control diet (C), control diet supplemented with 10% oligofructose (CF), diet enriched with hydrogenated vegetable fat (T) or diet enriched with hydrogenated vegetable fat supplemented with 10% oligofructose (TF). Diets were maintained during pregnancy and lactation. Serum TNF-α (tumor necrosis factor alpha) was assessed using a specific kit. Protein expression was determined by Western Blotting, and the relative mRNA levels were analyzed by RT-PCR (real-time polymerase chain reaction). We observed that 10% oligofructose supplementation during pregnancy and lactation increased offspring’s IL-6R (interleukin-6 receptor) mRNA levels in the liver and RET (retroperitoneal white adipose tissue) and decreased ADIPOR2 (adiponectin receptor 2) and ADIPOR1 (adiponectin receptor 1) gene expression in liver and EDL (extensor digital longus)/ SOL (soleus) muscles of CF group. Additionally, TF group presented with increased serum TNF-α, protein expression of p-NFκBp65 (phosphorylated form of nuclear factor kappa B p65 subunit) in liver and IL-6R mRNA levels in RET. These findings were accompanied by decreased levels of ADIPOR1 mRNA in the EDL and SOL muscles of the TF group. In conclusion, supplementing the dam’s diet with 10% of oligofructose during pregnancy and lactation, independent of hydrogenated vegetable fat addition, contributes to the increased pro-inflammatory status of 21-d-old offspring, possibly through the activation of the TLR4 (toll like receptor 4) pathway.

## Introduction

Maternal nutrition during pregnancy and lactation plays a fundamental role in the developing fetus and newborn until adulthood and may influence fetal "programming" through epigenetic modifications. Fetal "programming" is a phenomenon that is characterized by adaptive responses of the fetus to specific environmental conditions during critical periods of development, which may alter gene expression and permanently affect the structure and function of several organs and tissues, thereby inducing phenotypic changes [1–4].

Inadequate maternal nutrition during pregnancy and lactation may alter the physiologic and morphologic development of the fetus and newborn due to rapid cell division in these periods, thereby increasing the pup’s susceptibility to the development of metabolic diseases in adulthood [1–4]. A positive correlation between the development of metabolic diseases and the production of pro-inflammatory cytokines has been established [5,6].

Previously, in a Wistar rat model, our group demonstrated that the maternal intake of hydrogenated vegetable fats rich in trans fatty acids (TFA) during pregnancy and lactation seems to contribute to increased pro-inflammatory responses in the pups, leading to elevated TNF-α (tumor necrosis factor alpha) mRNA levels and TRAF-6 (TNF receptor-associated factor 6) protein expression in the adipose tissue. In addition, these findings were accompanied by a decrease in serum concentrations of adiponectin and ADIPOR1 (adiponectin receptor 1) protein levels in the adipose tissue of 21-d-old pups [7,8].

An experimental study by Pimentel et al. corroborated our results and showed increased serum endotoxin levels, increased NFκBp65 (nuclear factor kappa B p65 subunit), TLR4 (toll-like receptor 4) and MyD88 (myeloid differentiation primary response gene 88) protein expression, increased hypothalamic concentrations of IL-6 (interleukin-6), TNF-α and IL-1β (interleukin-1 beta) and decreased ADIPOR1 hypothalamic gene expression, thereby contributing to the hypothalamic inflammation of 90-d-old pups from Wistar dams that were fed a diet rich in TFA during pregnancy and lactation [9].

Hyperlipidemic diets may affect the "tight" cell junctions, thus harming the intestinal barrier and increasing the intestinal permeability to high molecular weight molecules such as lipopolysaccharides (LPS) [10,11]. LPS, also known as endotoxins, are found in the external cellular membranes of gram-negative intestinal bacteria. Elevated concentrations in the bloodstream have a potent immuno-stimulatory effect in the host [10,12,13]. LPS-induced TLR4 activation contributes to systemic inflammation by inducing the secretion of pro-inflammatory cytokines such as IL-6 and TNF-α [14].

In contrast, prebiotics and dietary fibers such as oligofructose (OF), fructooligosaccharides (FOS) and inulin appear to induce the production of short chain fatty acids (SCFA—acetate, propionate and butyrate), which have anti-inflammatory properties [15–17]. Studies have revealed that the end products of prebiotic microbial fermentation, particularly the SCFA, can modulate the host intestinal environment and alter intestinal permeability, thereby reducing LPS translocation to blood circulation [14,18,19].

Some studies have suggested that the maternal intestinal microbiota acts directly on the bacterial colonization and intestinal properties of newborns and infants; thus, the maternal intake of prebiotics and dietary fibers during pregnancy and lactation is considered important and beneficial for both the mother and the offspring well into later life [20]. Human maternal milk presents a large variety and amount of oligosaccharides which potentially prevent pathogen adhesion to the intestinal epithelium, influence the gut maturation process and the intestinal microbiota, and modify systemic functions in the offspring [21,22]. Recently, a few studies have demonstrated that some strains of bacteria present in the maternal gut can be transferred from the mother to the newborn through maternal milk [23].

In contrast, we previously showed that the maternal consumption of a control diet supplemented with OF (10%) during pregnancy and lactation is deleterious to offspring development and increases endotoxemia in 21-d-old pups [24]. Therefore, the objective of the present study was to evaluate the effect of a dietary supplementation of 10% OF during pregnancy and lactation in the presence or absence of hydrogenated vegetable fat on the pro-inflammatory status of 21-d-old male offspring.

## Materials and Methods

### Animals and treatments

The Ethic Research Committee/ Ethics Commission on Animal Use of the Universidade Federal de São Paulo (UNIFESP) approved all procedures for the care of the animals used in this study and followed the internationally recognized guidelines (CEUA protocol n°737014). Three-month-old female (16) and male (4) Wistar rats from CEDEME-UNIFESP (Centro de Desenvolvimento de Modelos Experimentais-UNIFESP) were used. The rats were kept under controlled conditions of light (12-h light/12-h dark cycle with lights on at 07:00) and temperature (24 ± 1 C) with *ad libitum* water and food. After an adaptation period of about 7 days, female rats (four animals in each experimental group) weighing approximately 225 g, were left overnight to mate, and copulation was verified the following morning by the presence of sperm in vaginal smears.

On the first day of pregnancy, the dams were isolated in individual polyethylene cages and sequentially divided into four groups, each receiving one of four diets: a control diet (C diet, C group), a control diet supplemented with oligofructose (CF diet, CF group), a diet enriched with hydrogenated vegetable fat (T diet, T group) or a diet enriched with hydrogenated vegetable fat supplemented with oligofructose (TF diet, TF group). The diets were maintained throughout pregnancy and lactation. At birth, the pups remained in the same group as the mother. The four diets were prepared according to the recommendations of the American Institute of Nutrition (AIN-93G) [25,26] and maintained a similar calorific and lipid content. The source of lipids for the C and CF diets was soybean oil, and the principal source for the T and TF diets was partially hydrogenated vegetable fat, which is rich in TFAs. The CF and TF diets were prepared by adding 100 g/kg of OF to the diet (Orafti P95, Pemuco, Chile). According to the manufacturer, the OF used in this study is a mixture of oligosaccharides extracted from chicory root. These oligosaccharides are composed of fructose units connected by β (2→1) links with a glucose unit terminating a few of these molecules. The DP of OF in this supplement ranges between 2 and 8. The centesimal composition of the diets is presented in Table 1. Pisani et al. previously described the fatty acid profile of the C and T diets [7].

**Table 1:** Composition of the control diet, control diet supplemented with oligofructose, diet enriched with trans fatty acids and diet enriched with trans fatty acids supplemented with oligofructose according to AIN-93.

			Diet (g/100g)	
Ingredient	C	CF	T	TF
Casein *	20.0	20.0	20.0	20.0
L-cystine †	0.3	0.3	0.3	0.3
Cornstarch †	62.0	52.0	62.0	52.0
Soybeanoil ‡	8.0	8.0	1.0	1.0
Hydrogenated vegetable fat $	-	-	7.0	7.0
Butylhydroquinone †	0.0014	0.0014	0.0014	0.0014
Mineral mixture §	3.5	3.5	3.5	3.5
Vitaminmixture #	1.0	1.0	1.0	1.0
Cellulose †	5.0	5.0	5.0	5.0
Cholinebitartrate †	0.25	0.25	0.25	0.25
Oligofructose £	-	10.0	-	10.0
Energy (kcal/g)	4.0	4.0	4.0	4.0

*Casein was obtained from Labsynth, São Paulo, Brazil.

^†^L-cystine, cornstarch, butylhydroquinone, cellulose and choline bitartrate were obtained from Viafarma, São Paulo, Brazil.

^‡^Oil was supplied from soybean (Lisa/Ind. Brazil).

^$^Hydrogenated vegetable fat was supplied from Unilever, São Paulo, Brazil.

^§^Mineral mix 9mg/kg diet): calcium, 5000; phosphorus, 1561; potassium, 3600;sodium, 1019; chloride, 1571; sulfur, 300; magnesium, 507; iron, 35; copper,6.0; manganese, 10.0; zinc, 30.0; chromium, 1.0; iodine 0.2; selenium, 0.15;fluoride, 1.00; boron, 0.50; molybdenum, 0.15; silicon, 5.0; nickel, 0.5; lithium,0.1; vanadium, 0.1 (AIN-93G, mineral mix, Rhoster, Brazil).

^#^Vitamin mix (mg/kg diet): thiamin HCL, 6.0, riboflavin, 6.0; pyridoxine HCL 7.0; niacin, 30.0; calcium pantothenate, 16.0; folic acid, 2.0; biotin, 0.2; vitamin B12,25.0; vitamin A palmitate 4000 IU; vitamin E acetate, 75; vitamin D3, 1000 IU; vitamin KI, 0.75. (AIN-93G, vitamin mix, Rhoster, Brazil).

^£^Oligofructose (P95) was manufactured by Orafti (Pemuco, Chile) and was obtained by Viafarma, São Paulo, Brazil.

On the day of delivery, considered day 0 of lactation, litter sizes were adjusted to eight pups each with an effort to maintain the largest possible number of males. Parturition was not induced and additional pups not used in this work were euthanized by decapitation after delivery. The offspring were weighed and measured weekly.

### Experimental procedures

In this study, only male pups were used for the molecular and biochemical analyses. In order to enable the comparison of the data with previous studies of our research group and to avoid any biochemical change in progeny, the male pups were euthanized by decapitation on postnatal day 21 in the specific laboratory room from 8 to 10 am. The animals were not fasted to avoid weaning stress and all possible efforts were made to minimize suffering of animals. Trunk blood was collected and then immediately centrifuged at 2500 rpm for 15 minutes. The serum was then separated and stored at −80°C for the determination of TNF-α. The retroperitoneal white adipose tissue (RET), liver, extensor digital longus (EDL) and soleus (SOL) muscles were isolated, immediately frozen in liquid nitrogen and stored at −80°C for the subsequent quantification of TLR4, TNFR1 (tumor necrosis factor receptor 1), IL-6R (interleukin-6 receptor), ADIPOR1 and ADIPOR2 (adiponectin receptor 2) mRNA and protein expressions. To perform these analyses was necessary to pooled tissues (RET, EDL and SOL) of different male pups from the same dam, partly due to the variation in body weight of the animals among experimental groups [24].

### Serum determination of TNF-α

The concentration of TNF-α in the serum was analyzed using the MILLIPLEX MAP 96-well Rat Cytokine/Chemokine Magnetic Bead Panel Kit (Millipore, Billerica, MA, USA). All procedures required for this analysis were performed by the Instituto Genese de Análises Científicas (IgAc, São Paulo, Brazil). The manufacturer’s recommendations, which are listed in the protocol accompanying the product, were followed for the analysis. The assay did not detect the concentrations of IL-6 and IL-10 (interleukin-10) cytokines; thus, only the TNF-α serum concentration was presented in the results.

### RNA extraction and real-time polymerase chain reaction (RT-PCR)

Total RNA from tissues was extracted with Tri-Reagent (Sigma, St. Louis, MO, USA) according to the manufacturer's recommendations. The total RNA concentration per microliter was measured using a spectrophotometer, NanoDrop ND-1000 (NanoDrop Technologies Inc., Wilmington, DE, USA), and the readings were acquired at wavelengths of 260, 280 and 230 nm. The purity was estimated by the 260/280 nm ratio, which must range between 1.8 and 2.0 for nucleic acids. All samples were maintained at −80°C.

The TLR4, TNFR1, IL6R, ADIPOR1 and ADIPOR2 mRNA from the RET, liver, EDL and SOL were quantified by real time RT-PCR. Two micrograms of each total RNA sample was reverse transcribed using an M-MLV Reverse Transcriptase kit (PROMEGA, Madison, WI, USA), and cDNA was synthesized to a final volume of 20 μL. The relative levels of TLR4, TNFR1, IL6R, ADIPOR1 and ADIPOR2 mRNA were quantified in real time using a SYBR Green primer in an ABI Prism 7500 Sequence Detector (both from Applied Biosystems, Foster City, CA, USA). The relative levels of the housekeeping gene hypoxanthine phosphoribosyltransferase (HPRT) were measured. The primers used were TLR4 5’-GCA TCA TCT TCA TTG TCC TTG AGA-3’ (sense) and 5’-CTA CCT TTT CGG AAC TTA GGT CTA CT-3’ (antisense), TNFR1 5’-GAA CAC CGT GTG TAA CTG CC-3’ (sense) and 5’-ATT CCT TCA CCC TCC ACC TC-3’ (antisense), IL6R 5’-AAG CAG GTC CAG CCA CAA TGT AG-3’ (sense) and 5’-CCA ACT GAC TTT GAG CCA ACG AG-3’ (antisense), ADIPOR1 5’-AAT CCT GCC CAG TCA TGA AG-3’ (sense) and 5’-CAT CTC CTG GGT CAG CCT TA-3’ (antisense), ADIPOR2 5’-ATG TTT GCC ACC CCT CAG TA-3’ (sense) and 5’-CAG ATG TCA CAT TTG GCA GG-3’ (antisense), and hypoxanthine phosphoribosyltransferase 5’-CTC ATG GAC TGA TTA TGG ACA GGA C-3’ (sense) and 5’-GCA GGT CAG CAA AGA ACT TAT AGC C-3’ (antisense).

The results were obtained using the Sequence Detector software (Applied Biosystems) and were expressed as the relative increase using the method of 2^-ΔΔCt^ described by Livak and Schmittgen [27].

### Protein analysis by Western Blotting

After euthanasia, the RET and liver were removed and placed in 800 ¼L of extraction buffer prepared on the day of experiment (100 mM Trizma base pH 7.5, 20 mM EDTA, 100 mM sodium fluoride, 100 mM sodium pyrophosphate, 10 mM sodium orthovanadate, 2 mM PMSF-phenylmethylsulfonyl fluoride and 0.1 mg of aprotinin per mL). The tissues were rapidly homogenized using a polytron, and 800 ¼L of 1% Triton X-100 was then added. After 30 minutes, the homogenate was centrifuged at 14000 rpm for 40 minutes at 4°C. The supernatant was maintained on ice, and the total protein content was determined by the Bradford method using the Bio-Rad reagent (Bio-Rad Laboratories, Hercules, CA, USA) with bovine serum albumin (BSA) as reference.

The samples were treated with Laemmli buffer (0.01% bromophenol blue, 100 mM sodium phosphate pH 7.0, 50% glycerol, 10% SDS) at a ratio of 4:1 containing 100 mM DTT (dithiothreitol). The proteins (50μg) were boiled for 5 min before loading onto 10% SDS-PAGE in a Bio-Rad miniature slab gel apparatus (Bio-Rad, Hercules, CA, USA). Electrotransfer of proteins from the gel to the nitrocellulose membrane was performed for ~90 minutes/4 gels at 15 V (constant) in a Bio-Rad semi-dry transfer apparatus (Bio-Rad, Hercules, CA, USA). Nonspecific protein binding to the nitrocellulose membrane was reduced by overnight pre-incubation at 22°C in blocking buffer composed of basal solution (100 mM Trizma base pH 7.5, 500 mM NaCl, Tween 20 0.02%) containing 1% BSA.

The nitrocellulose membranes were incubated overnight at 22°C with antibodies against TLR4, NFĸB p50 in the phosphorylated form (p-NFĸB p50), IL-6Rα and ADIPOR1 (Santa Cruz Biotechnology, CA, USA) and NFĸB p65 in the phosphorylated form (p-NFĸB p65), α-Tubulin and β-Tubulin (Cell Signaling Technology, Inc., MA, USA). Antibodies were diluted in 1:1000 (Santa Cruz) or 1:5000 (Cell Signaling) with blocking buffer and then washed for 30 min in basal solution. The blots were subsequently incubated with a peroxidase-conjugated secondary antibody for 1 h at 22°C. To evaluate protein loading, membranes were stripped and reblotted with an anti-α-tubulin (RET) or anti-β-tubulin (liver) antibody as appropriate. Specific bands were detected by chemiluminescence following ECL reagent addition (Amersham/GE), and the visualization/capture was performed by exposure to Alliance 4.7 equipment (Uvitec, Cambridge, UK). Band intensities were determined by optical densitometry (Scion Image-Release Beta 3b, NIH, USA).

### Statistical analysis

Data were submitted to Shapiro-Wilk (normality) and Levenne (homogeneity) quality tests and then standardized to the Z score, if necessary. The statistical significance of the differences among the means of the four groups was assessed using a two-way analysis of variance (ANOVA) followed by a Bonferroni post hoc test. All statistical tests were performed using the PASW Statistics 18 program, except for the comparison between the C and TF groups, which was performed in the Stats Direct program using a one-way ANOVA and Bonferroni post hoc test. The other functions were executed using the Microsoft Excel 2010 program. All results are presented as the means ± standard error of the mean (SEM), and differences were considered to be significant when *p*≤ 0.05. Grubb's test (GraphPad Software, Inc., La Jolla, CA, USA) was performed to remove significant outliers.

## Results

**Fig 1:**
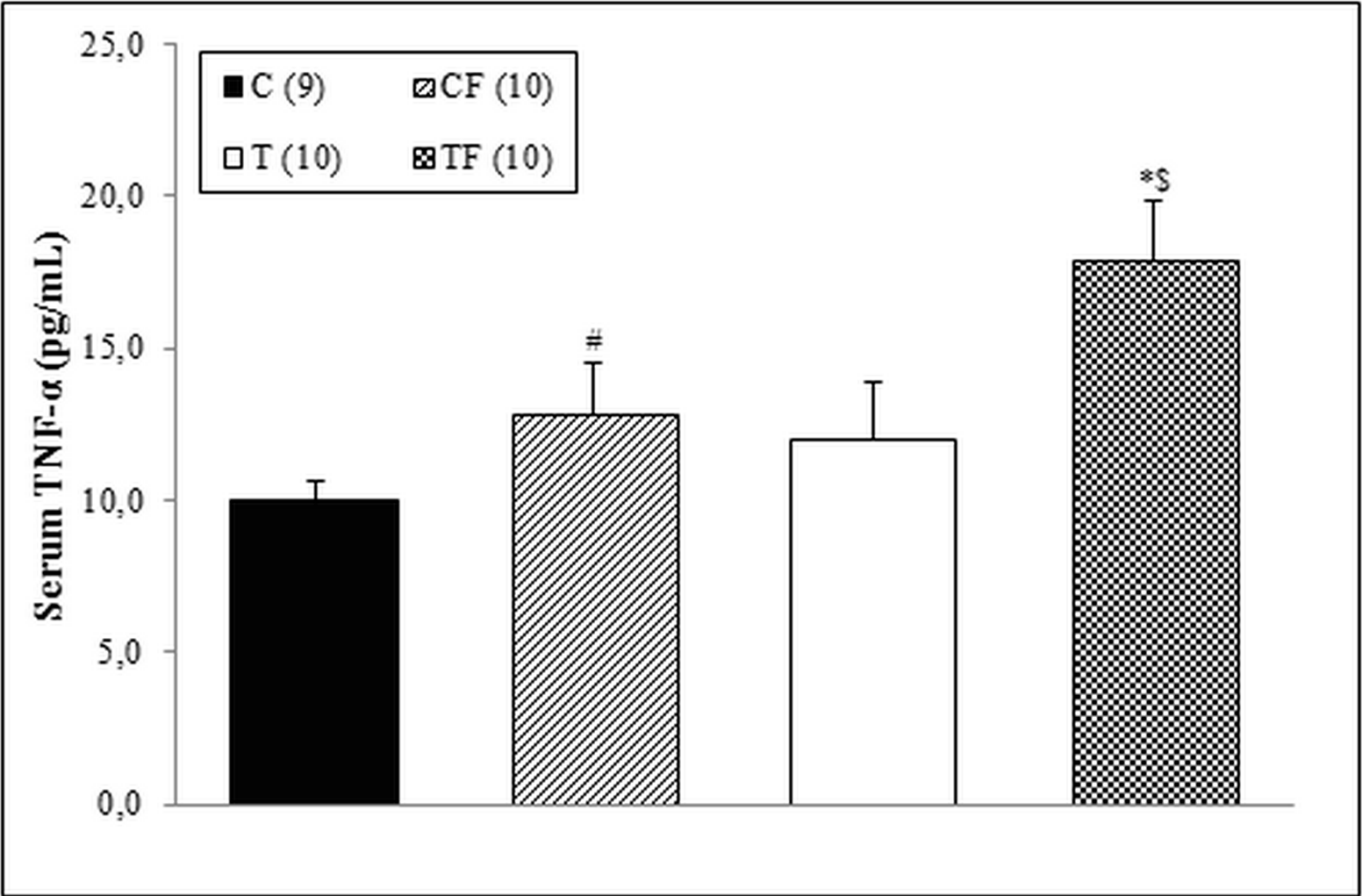
Serum concentration of TNF-α. C—male offspring of dams fed control diet; CF—male offspring of dams fed control diet supplemented with oligofructose; T—male offspring of dams fed diet enriched with hydrogenated vegetable fat; TF—male offspring of dams fed diet enriched with hydrogenated vegetable fat supplemented with oligofructose. The number in parentheses refers to the sample value (C group presented one outlier sample). Data are means ± SEMs. *p≤ 0.05 versus C. ^$^p≤ 0.05 versus T. ^#^p≤ 0.05 versus TF.

In order to investigate the effects of maternal supplementation with 10% of oligofructose during pregnancy and lactation in the presence or absence of hydrogenated vegetable fat on the pro-inflammatory status of 21-d-old male offspring, we evaluated the TNF-α serum concentration, mRNA and protein levels of receptors involved in the inflammatory process (TLR4, TNFR1, IL-6R and ADIPOR1/ADIPOR2) of different tissues and protein expression of phosphorylated form of transcriptional factor NFκB (p50 and/or p65 subunits) in liver and RET. The results demonstrated a higher serum concentration of TNF-α in the TF group when compared with the T (*p*<0.05), C (*p*<0.01) and CF (*p*<0.05) groups (Fig 1).

**Fig 2:**
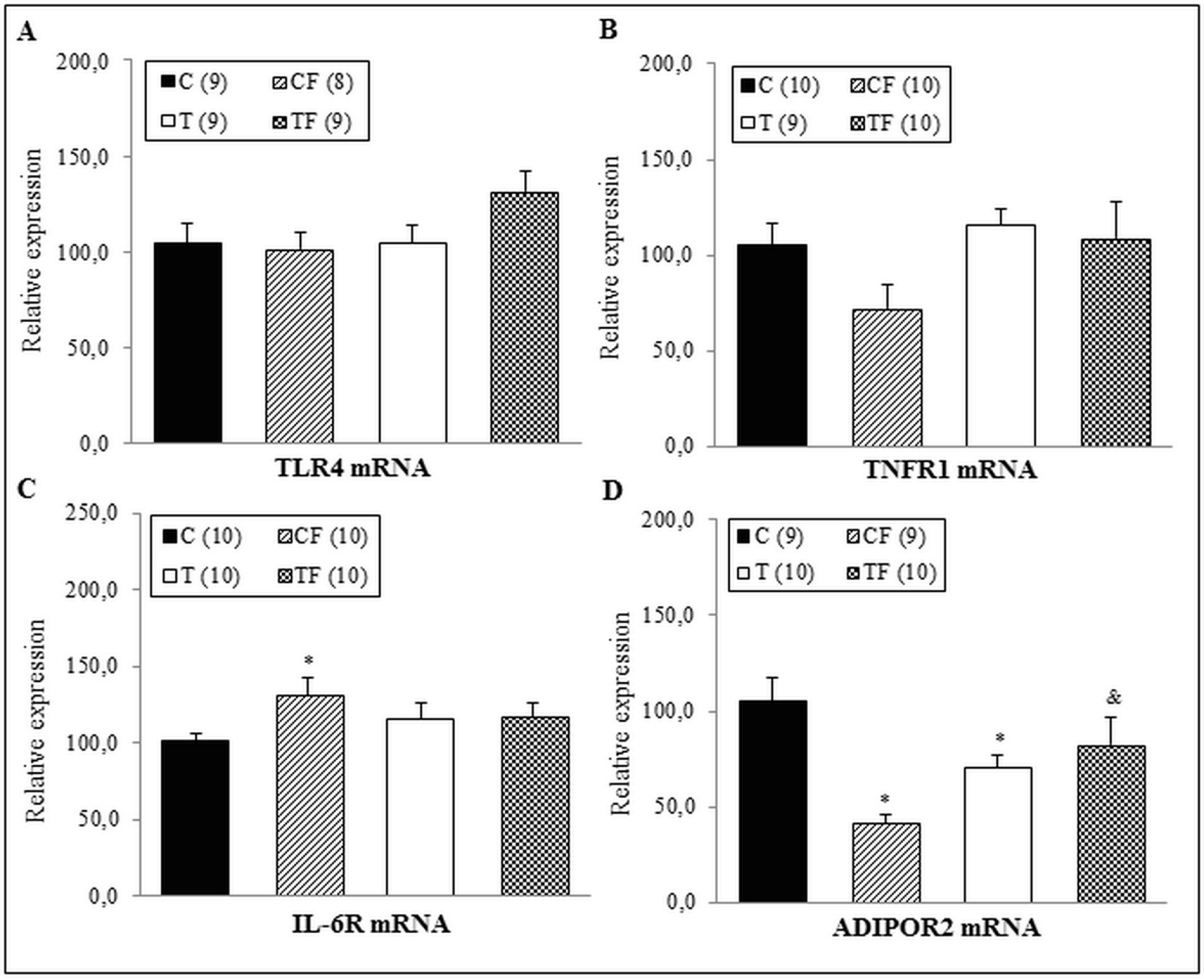
(A) Gene expression of TLR4 in the liver, (B) Gene expression of TNFR1 in the liver, (C) Gene expression of IL-6R in the liver and (D) Gene expression of ADIPOR2 in the liver. C—male offspring of dams fed control diet; CF—male offspring of dams fed control diet supplemented with oligofructose; T—male offspring of dams fed diet enriched with hydrogenated vegetable fat; TF—male offspring of dams fed diet enriched with hydrogenated vegetable fat supplemented with oligofructose. The number in parentheses refers to the sample value (Fig 2A and 2D: CF group presented one outlier sample). Data are means ± SEMs. Results are expressed in arbitrary units, stipulating 100 as the control value. *p≤ 0.05 versus C. ^&^p≤ 0.05 versus CF.

Regarding gene expressions of TLR4, TNFR1, IL-6R and ADIPOR2 in liver, we observed that the IL-6R mRNA levels of CF group was higher than that of the C group (+29.1%; *p*<0.05) (Fig 2C). The ADIPOR2 mRNA levels in the CF (-61.2%) and T (-33.5%) groups were significantly lower in relation to the C group (*p*<0.001 and *p*<0.05, respectively). In parallel, the TF group had increased ADIPOR2 gene expression compared with the CF group (+100.6%; *p*<0.01) (Fig 2D). However, TLR4 and TNFR1 mRNA levels did not differ among the C, CF, T and TF groups (Fig 2A and 2B, respectively).

**Fig 3:**
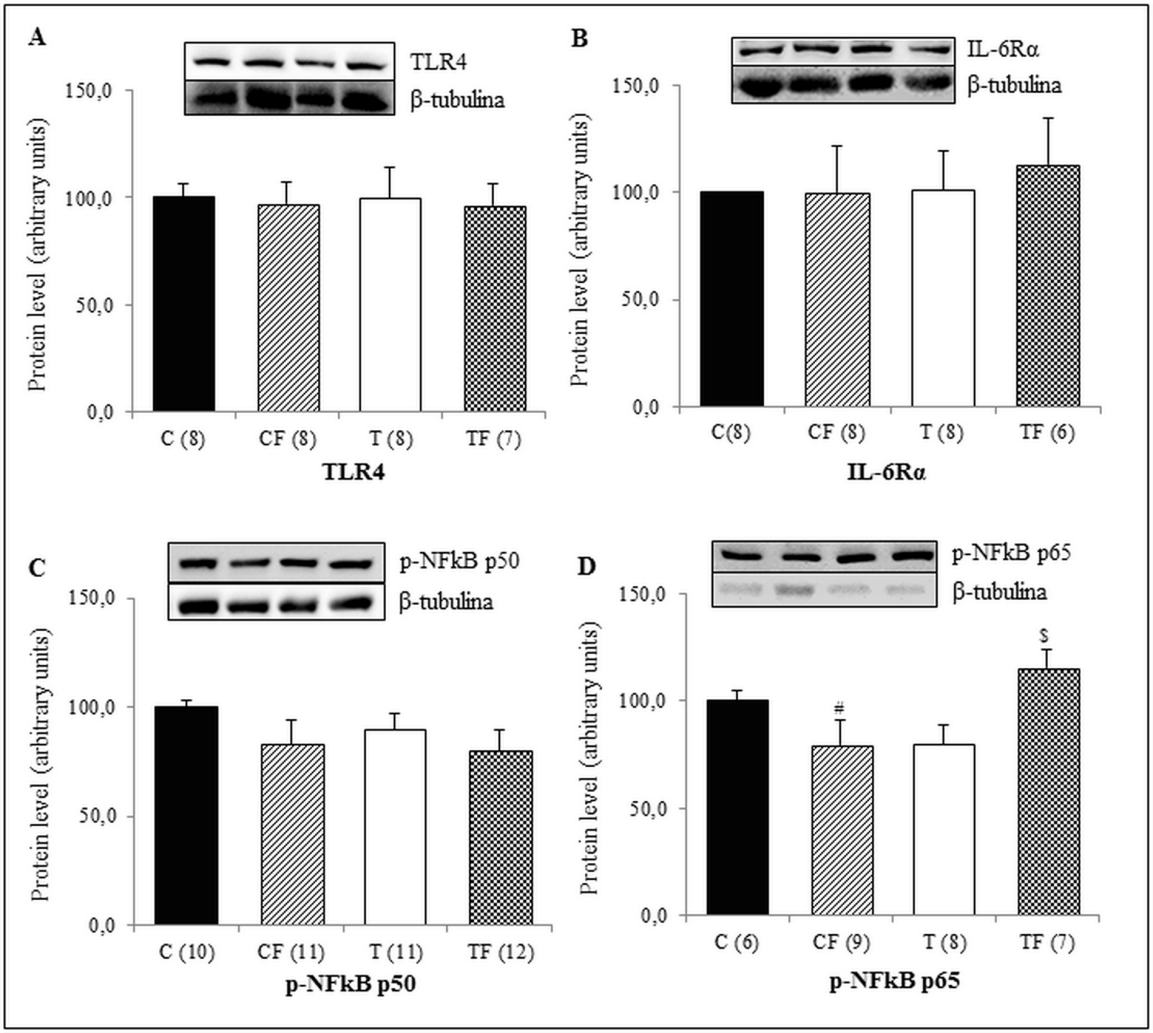
(A) Protein expression of TLR4 in the liver, (B) Protein expression of IL-6Rα in the liver, (C) Protein expression of NFκB p50 phosphorylated form in the liver (p-NFκB p50) and (D) Protein expression of NFκB p65 phosphorylated form in the liver (p-NFκB p65). C—male offspring of dams fed control diet; CF—male offspring of dams fed control diet supplemented with oligofructose; T—male offspring of dams fed diet enriched with hydrogenated vegetable fat; TF—male offspring of dams fed diet enriched with hydrogenated vegetable fat supplemented with oligofructose. The number in parentheses refers to the sample value (Fig 3B and 3D: TF group presented one outlier sample). Data are means ± SEMs. Results are expressed in arbitrary units, stipulating 100 as the control value. ^$^p≤ 0.05 versus T. ^#^p≤ 0.05 versus TF.

We also observed that the protein expression of p-NFκB p65 in liver of TF group was significantly higher than the T (+45.3%; *p*<0.05) and CF (+45.1%; *p*<0.05) groups (Fig 3D). However, protein levels of TLR4, IL-6Rα and p-NFκB p50 in this tissue were unchanged among the groups of 21-d-old pups (Fig 3A, 3B and 3C, respectively).

**Fig 4:**
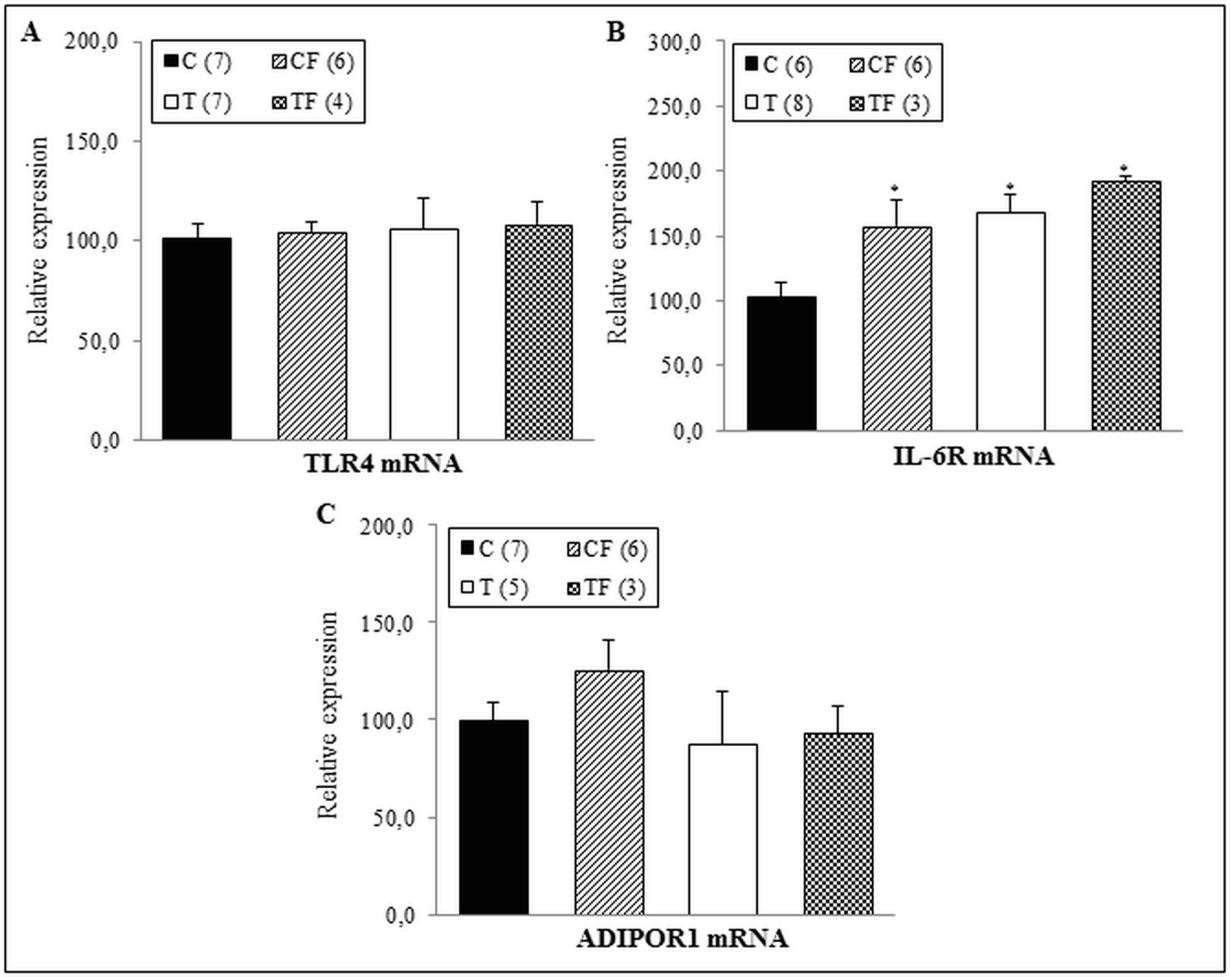
(A) Gene expression of TLR4 in the RET, (B) Gene expression of IL-6R in the RET and (C) Gene expression of ADIPOR1 in the RET. C—male offspring of dams fed control diet; CF—male offspring of dams fed control diet supplemented with oligofructose; T—male offspring of dams fed diet enriched with hydrogenated vegetable fat; TF—male offspring of dams fed diet enriched with hydrogenated vegetable fat supplemented with oligofructose. The number in parentheses refers to the sample value (Fig 4B: TF group presented one outlier sample). Data are means ± SEMs. Results are expressed in arbitrary units, stipulating 100 as the control value. *p≤ 0.05 versus C.

When the mRNA levels of TLR4, IL-6R and ADIPOR1 were analyzed in the offspring’s RET, we observed an IL-6R gene expression increased in the CF (+52.6%; *p*<0.05), T (+62.6%; *p*<0.01) and TF (+86.5%; *p*<0.001) groups compared with C group (Fig 4B). However, gene expression of TLR4 and ADIPOR1 was similar among the studied groups (Fig 4A and 4C, respectively).

**Fig 5:**
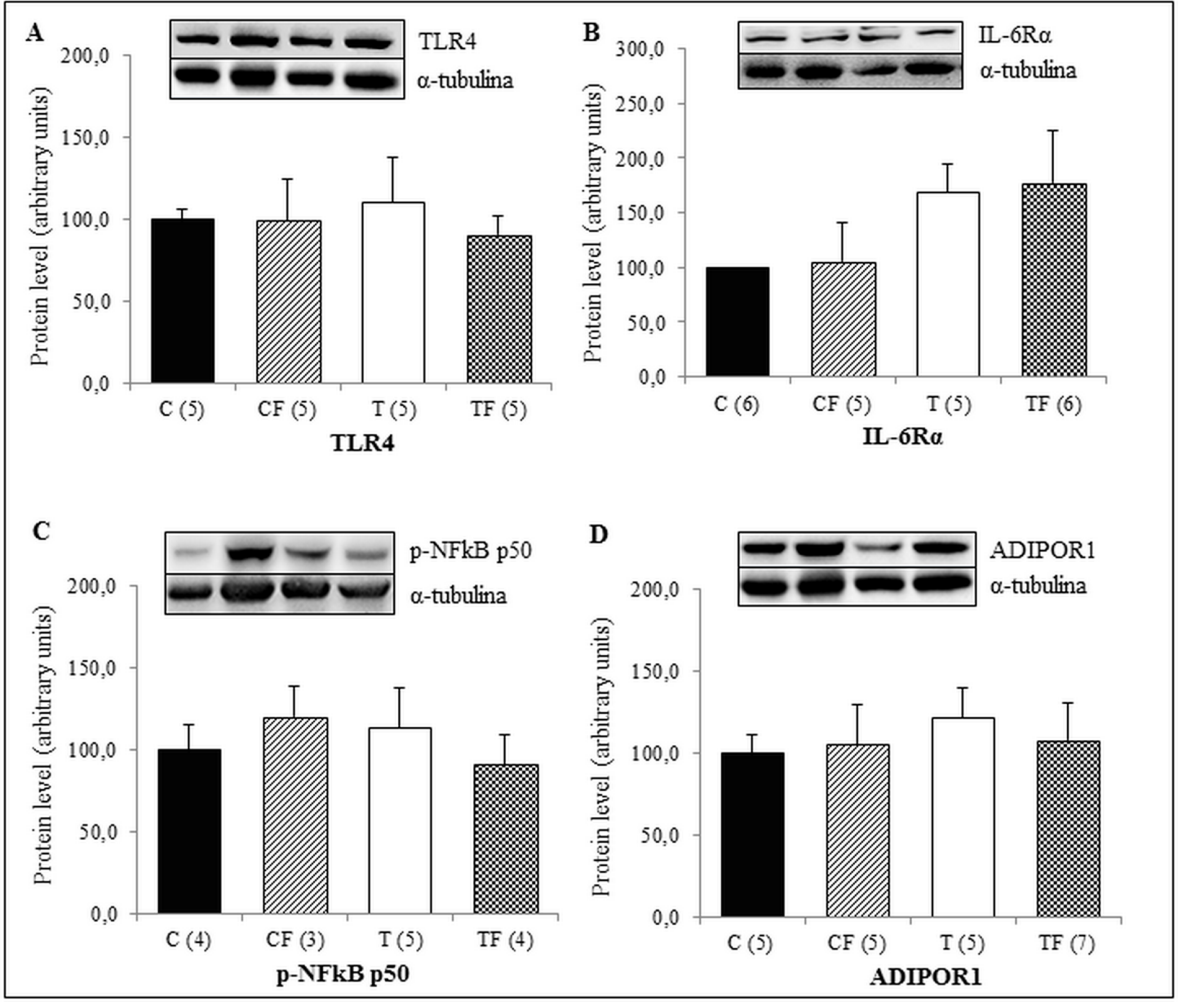
(A) Protein expression of TLR4 in the RET, (B) Protein expression of IL-6Rα in the RET, (C) Protein expression of NFκB p50 phosphorylated form in the RET (p-NFκB p50) and (D) Protein expression of ADIPOR1 in the RET. C—male offspring of dams fed control diet; CF—male offspring of dams fed control diet supplemented with oligofructose; T—male offspring of dams fed diet enriched with hydrogenated vegetable fat; TF—male offspring of dams fed diet enriched with hydrogenated vegetable fat supplemented with oligofructose. The number in parentheses refers to the sample value (Fig 4D: T group presented one outlier sample). Data are means ± SEMs. Results are expressed in arbitrary units, stipulating 100 as the control value.

There were no differences among the experimental groups in the RET protein levels of TLR4, IL-6Rα, p-NFκB p50 and ADIPOR1 (Fig 5).

**Fig 6:**
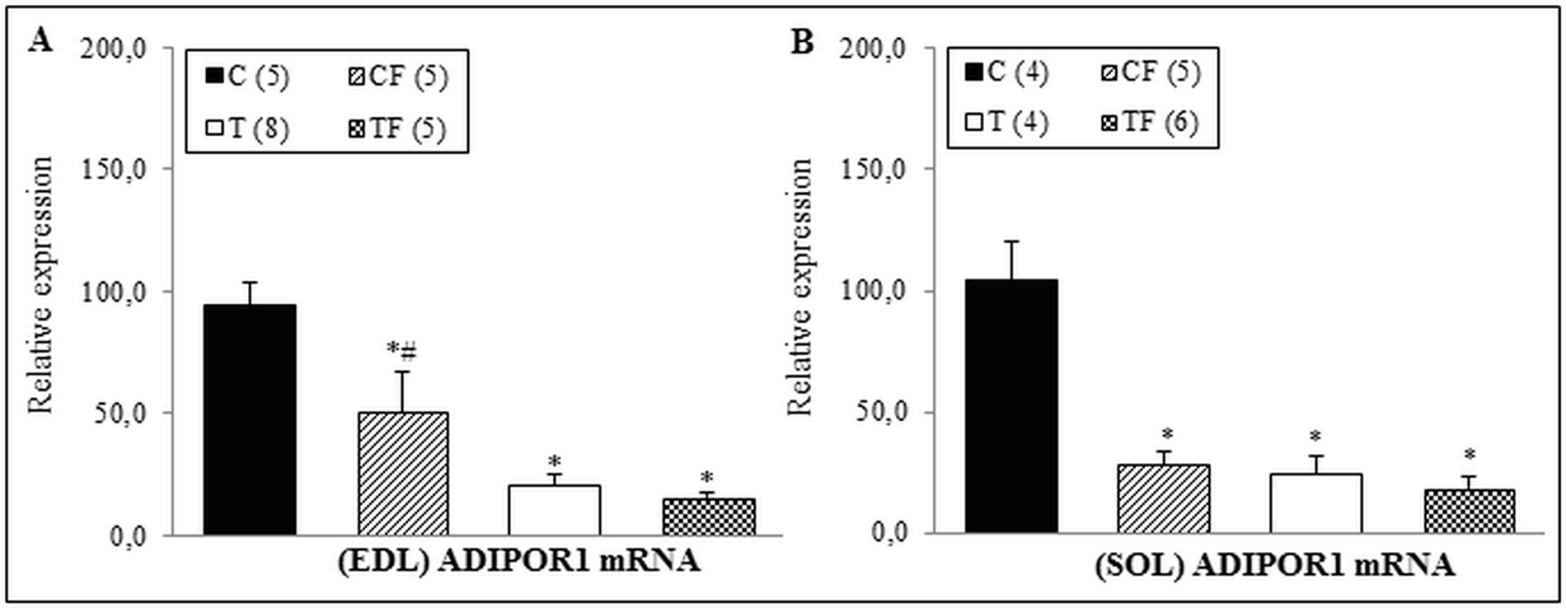
(A) Gene expression of ADIPOR1 in the EDL muscle and (B) Gene expression of ADIPOR1 in the SOL muscle. C—male offspring of dams fed control diet; CF—male offspring of dams fed control diet supplemented with oligofructose; T—male offspring of dams fed diet enriched with hydrogenated vegetable fat; TF—male offspring of dams fed diet enriched with hydrogenated vegetable fat supplemented with oligofructose. The number in parentheses refers to the sample value. Data are means ± SEMs. Results are expressed in arbitrary units, stipulating 100 as the control value. *p≤ 0.05 versus C. ^#^p≤ 0.05 versus TF.

Hoping to complement the pro-inflammatory results presented in liver and RET of 21-d-old offspring we determinate the ADIPOR1 mRNA levels in two different muscles. In EDL muscle, the findings showed a significantly decrease in ADIPOR1 mRNA levels of the CF (-46.0%; *p*<0.01), T (-78.0%; *p*<0.001) and TF (-83.3%; *p*<0.001) groups when compared with C group. Additionally, the gene expression of ADIPOR1 in the CF group was higher than that of the TF group (+223.1%; *p*<0.05) (Fig 6A).

Accordingly, in the SOL muscle, the gene expression of ADIPOR1 in the CF (-72.6%; *p*<0.001), T (-76.3%; *p*<0.001) and TF (-82.6%; *p*<0.001) groups was lower than that of the C group (Fig 6B).

## Discussion

The present results indicate that supplementing the dam’s diet with this dosage of oligofructose (10%) during pregnancy and lactation contributes to an increased pro-inflammatory status in the 21-d-old offspring.

In this study, the 10% OF supplementation during pregnancy and lactation increased the TNF-α serum concentration, the p-NFκB p65 protein expression in the liver and IL-6R mRNA in the liver and RET. These findings were accompanied by decreased ADIPOR2 mRNA in the liver and reduced ADIPOR1 mRNA levels in the EDL and SOL muscles of the 21-d-old pups.

Previous studies have shown that the inulin-type fructans selectively stimulate the growth and activity of determinate bacteria present in the colonic microbiota, thereby modulating the intestinal environment by changing intestinal permeability, bacterial composition and SCFA production and contributing to the decreased LPS serum concentration [14,28,29]. In fact, Dehghan et al. showed that the administration of 10 g/d oligofructose-enriched inulin for 8 weeks decreased the serum concentrations of LPS, IL-6 and TNF-α in patients with type 2 diabetes [30]. Moreover, Cani et al. reported that mice fed a high-fat diet combined with OF during 14 weeks presented a decrease in endotoxemia and IL-6 plasma levels [13].

However, in a recent study, we showed an increased LPS serum concentration and genomic DNA levels of lactobacillus spp. in colonic feces from the CF pups, indicating that the maternal intake of a diet supplemented with 10% OF during pregnancy and lactation may lead to changes in intestinal permeability and in bacterial composition, possibly influencing endotoxemia and the LPS-stimulated inflammatory responses in the 21-d-old offspring [24]. In fact, we also observed that diet supplementation with oligofructose (10%) during pregnancy and lactation caused diarrhea in dams (CF and TF groups) and changed the maternal body weight and food intake among the four experimental groups (data not shown).

In agreement, the present study showed an increase in IL-6R mRNA levels both in the liver and RET of the CF group (Figs 2C and 4B, respectively); without changing IL-6Rα protein expression (Figs 3B and 5B, respectively). Complementary, Hachul et al. observed that the TNF-α liver content of 21-d-old offspring was greater in the CF group than the C group [31]. It is well established that the genetic information is transcribed from DNA to mRNA, which subsequently serves as a mold for the translation process, thereby forming the proteins [32]. Therefore, herein we hypothesized that the translation of IL-6Rα mRNA may still not have been started in the 21-d-old pups or the differences in the translation-mediated protein level of IL-6Rα may still not be significantly displayed among experimental groups. These two possibilities appear to indicate a possible long-term augment in the IL-6Rα protein levels of offspring.

Although only pups from the CF group showed elevated serum endotoxemia in a previous study [24], in this study, the 21-d-old offspring of the TF group presented with higher p-NFκB p65 protein levels in the liver (Fig 3D) and an increased serum concentration of TNF-α (Fig 1). Gene and protein expressions of TLR4 were not altered in different tissues (Figs 2A, 3A, 4A and 5A); however it is known that the TLR4 signaling pathway includes the NF-κB activation [33], which requires the NF-κB p65 subunit phosphorylation and others post-translational modifications [34].

Furthermore, the TF and T groups also showed increased IL-6R mRNA levels in the RET (Fig 4B) compared to C group. Accordingly, in a previous study, we also found that OF supplementation (10%) during pregnancy and lactation increased IL-6 and TNF-α contents in adipose tissue of the TF group compared with the C group and was accompanied by a decreased adiponectin serum concentration in the 21-d-old offspring of the TF, T and CF groups [31].

Taken together, the mentioned results suggest that the signaling cascade of TLR4 may have been activated in pups that were exposed to 10% OF and TFA during pregnancy and lactation, independent of the increased LPS serum concentration.

Dietary nutrients (e.g., prebiotic fibers and fatty acids) seem to interfere directly with the host’s gut microbiota composition and health status [13, 35]. In fact, Semova et al. demonstrated in zebrafish that the presence of gut microbiota stimulates intestinal dietary fatty acid (FA) absorption and lipid droplet (LD) formation—for temporary fat storage—in the gut epithelium. Additionally, gut microbes appear to induce the accumulation of dietary FAs in extra-intestinal tissues such as the liver [36]. Further, same authors have suggested that diet-induced changes in microbiota composition can influence dietary FA absorption [36].

Thus, we hypothesize that the dam’s consumption of a high-OF diet (10%) enriched with TFA during pregnancy and lactation may detrimentally alter the gut microbial community of the 21-d-old offspring, leading to an increase in the intestinal absorption of dietary FAs [36] such as saturated fatty acids (SFA). Similar to LPS, SFA are involved in the TLR4 pathway activation through the activation of the transcription factor NFκB (p50 and p65), which favors the production of pro-inflammatory cytokines such as TNF-α and IL-6 [33, 37, 38]. Thus, in the bloodstream and extra-intestinal tissues, these absorbed FAs are likely involved in the activation of the TLR4 pathway, thereby negatively influencing the pro-inflammatory status of the TF group pups.

In association with the pro-inflammatory process observed in offspring, both the CF and TF groups presented with decreased ADIPOR1 and ADIPOR2 gene expression in different tissues, including liver and EDL/SOL muscles (Figs 2D, 6A and 6B). However, in RET there was no differences in gene and protein expressions of ADIPOR1 (Figs 4C and 5D, respectively), suggesting that the inflammatory response in the 21-d-old offspring may be tissue-specific.

Adiponectin is abundantly released by adipose tissue, exerts its function through the receptors ADIPOR1 and ADIPOR2 and has anti-atherosclerotic and anti-inflammatory properties because it inhibits the expression of vascular adhesion molecules and pro-inflammatory cytokines [39]. Zhou et al. demonstrated that adiponectin mRNA was poorly expressed but that IL-6, TNF-α and TLR4 mRNA were highly expressed in epicardial adipose tissue of patients with coronary artery disease [40]. Similarly, Ajuwon and Spurlock reported that adiponectin down-regulates NFκB activation by LPS and reduces the LPS-mediated increase in IL-6 and TNF-α mRNA expression in pig adipocytes [41]. Furthermore, Wulster-Radcliffe et al. showed that pre-treatment with adiponectin disrupts LPS-induced NFκB nuclear translocation in porcine blood-derived macrophages [42]. Likewise, Lira et al. found a decrease in IL-6 levels and both NFκB p50 and NFκB p65 nuclear activity in 3T3-L1 adipocytes treated with adiponectin in the presence of LPS [43]. Additionally, the adiponectin level and ADIPOR1/ADIPOR2 expression are reduced in obesity and obesity-related diseases and likely contribute to the characteristic low-grade inflammation because of the absence of the anti-inflammatory effects of adiponectin [39].

The findings described in the present study reveal that the pro-inflammatory responses could be influenced by the amount and type of prebiotics and fatty acids ingested and by the treatment period and the individual physiological conditions.

## Conclusion

In conclusion, supplementing the dam’s diet with 10% oligofructose during pregnancy and lactation in the presence or absence of hydrogenated vegetable fat contributes to the increase in the pro-inflammatory status of 21-d-old offspring. In particular, the isolated supplementation of the maternal diet with oligofructose (10%) seems to activate the LPS-mediated TLR4 pathway. In contrast, high oligofructose supplementation with hydrogenated vegetable fat during pregnancy and lactation triggered inflammatory tissue-specific responses in the offspring, likely through the activation of the TLR4 pathway but independent of the elevation in the pup’s serum endotoxin levels, increased pro-inflammatory cytokine production and decreased the adiponectin levels. It is still precipitate the generalization of present findings to other animal species, including humans. Further studies should investigate the dose-response effect of oligofructose supplementation during pregnancy and lactation on the development, metabolism, endotoxemia and inflammatory mechanisms in pups.

## Supporting Information

S1 ARRIVE Checklist ARRIVE Guidelines Checklist. (DOC)
