# Supplementary material for: Maternal Supplementation with Oligofructose (10%) during Pregnancy and Lactation Leads to Increased Pro-Inflammatory Status of the 21-D-Old Offspring
Source: PLoS One. 2015 Jul 6;10(7):e0132038. doi: 10.1371/journal.pone.0132038 (PMC4493056; doi:10.1371/journal.pone.0132038)

**S1 ARRIVE Checklist. ARRIVE Guidelines Checklist**


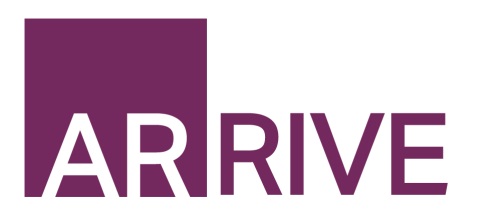


The ARRIVE Guidelines Checklist

Animal Research: Reporting In Vivo Experiments

Carol Kilkenny^1^, William J Browne^2^, Innes C Cuthill^3^, Michael Emerson^4^ and Douglas G Altman^5^

*^1^The National Centre for the Replacement, Refinement and Reduction of Animals in Research, London, UK, ^2^School of Veterinary Science, University of Bristol, Bristol, UK, ^3^School of Biological Sciences, University of Bristol, Bristol, UK, ^4^National Heart and Lung Institute, Imperial College London, UK, ^5^Centre for Statistics in Medicine, University of Oxford, Oxford, UK.*

|  | ITEM | RECOMMENDATION | Section/ Paragraph | |
| --- | --- | --- | --- | --- |
| 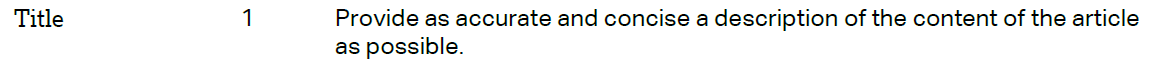 | | | Page 1 | |
| 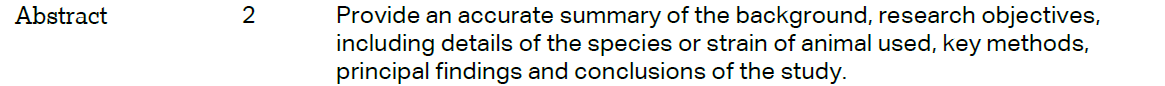 | | | Pages 2-3  Abstract | |
| INTRODUCTION | | |  | |
| 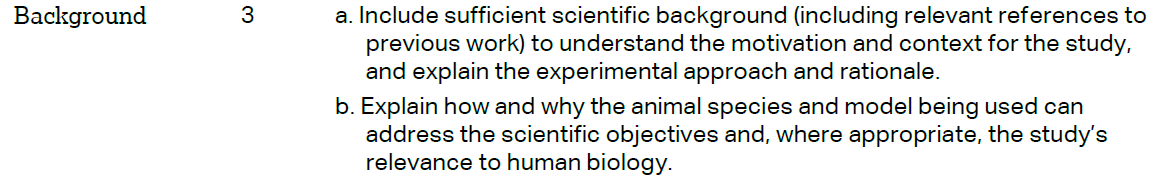 | | | Pages 4-6  Introduction | |
| 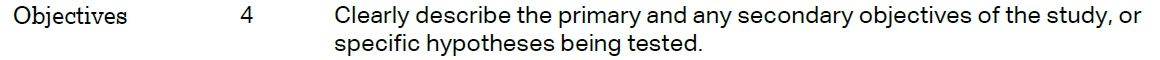 | | | Page 6  Introduction | |
| METHODS | | |  | |
| 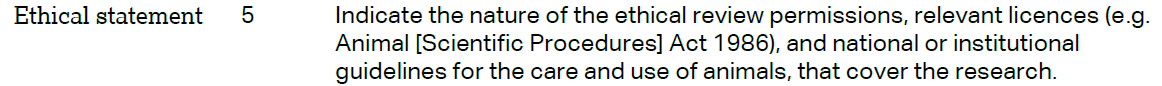 | | | Page 6  Materials and Methods | |
| 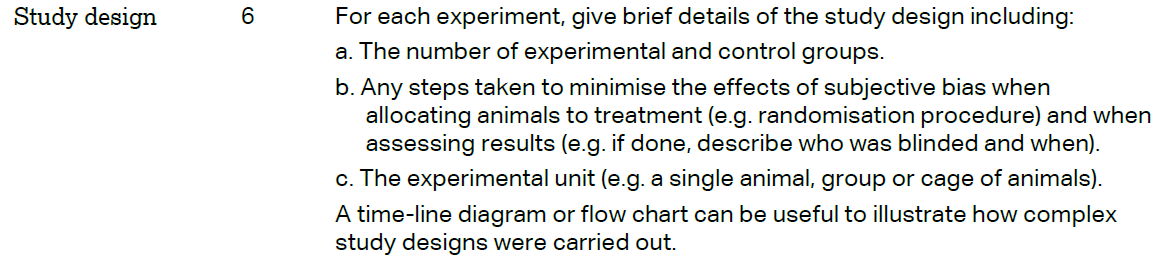 | | | Page 7  Materials and Methods | |
| 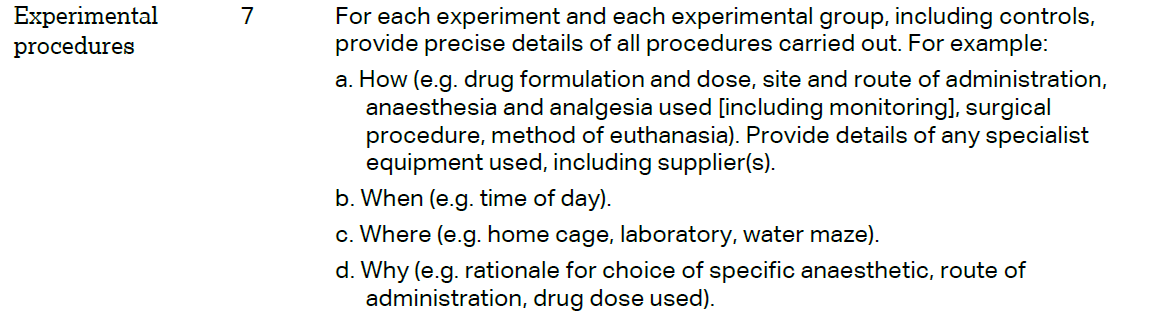 | | | Pages 8-9  Materials and Methods | |
| 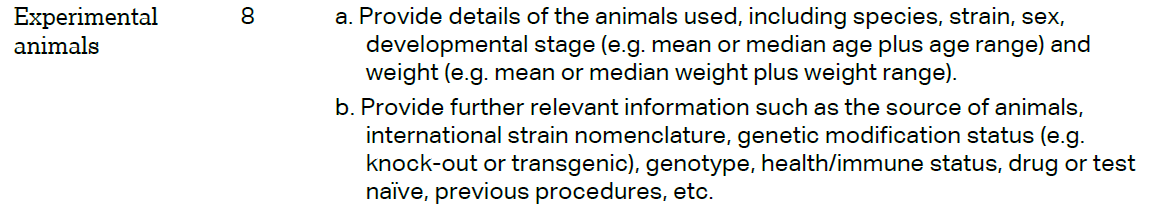 | | | Page 6  Materials and Methods | |
| 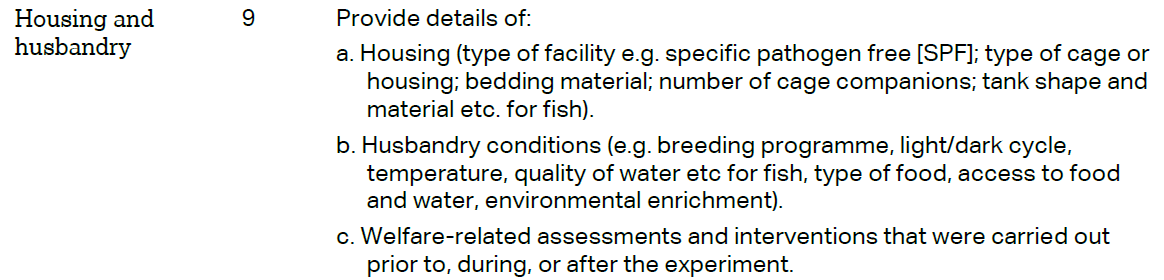 | | | Pages 6-8  Materials and Methods | |
| 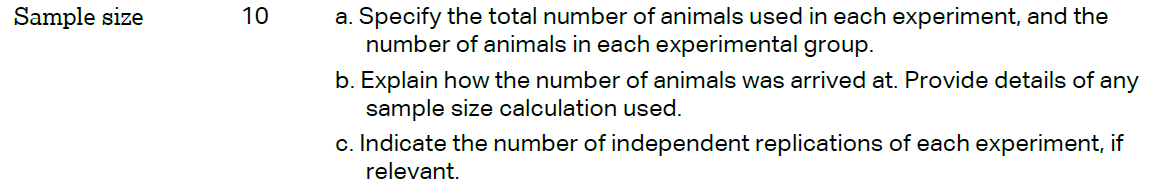 | | | Page 6 and 8  Materials and Methods | |
| 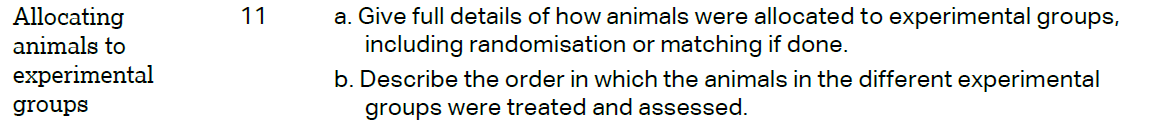 | | | Page 7-8  Materials and Methods | |
| 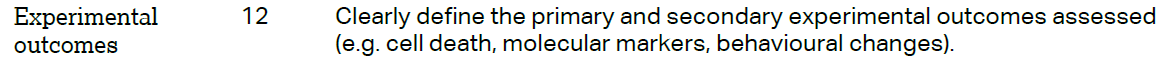 | | | Defined in experimental model | |
| 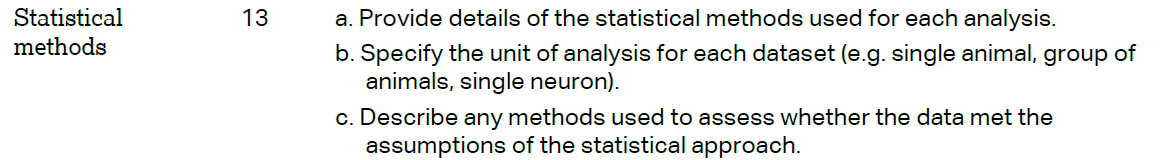 | | | Page 12 and 13  Materials and Methods | |
| RESULTS | | |  | |
| 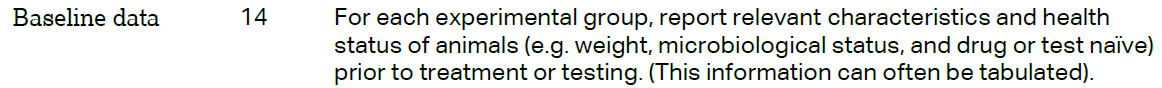 | | | The animal characteristics is described in result topic | |
| 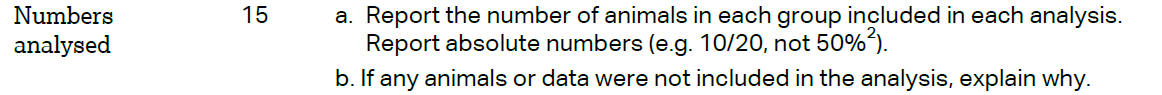 | | | The number of animals in each experimental group is shown in the figure (Fig. 1, Fig. 2, Fig. 3, Fig. 4, Fig. 5 and Fig. 6) of each parameter analyzed.  Results  Page 12 and 13  Materials and Methods | |
| 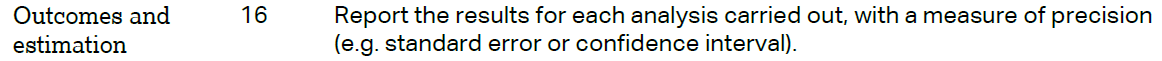 | | | Pages 13-16  Results | |
| 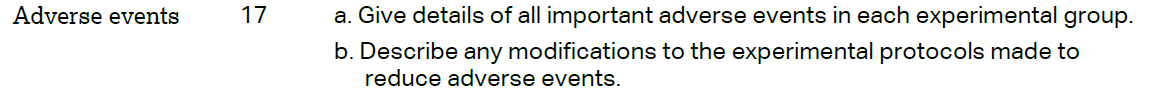 | | | Had not important adverse events in experimental group | |
| DISCUSSION | | |  | |
| 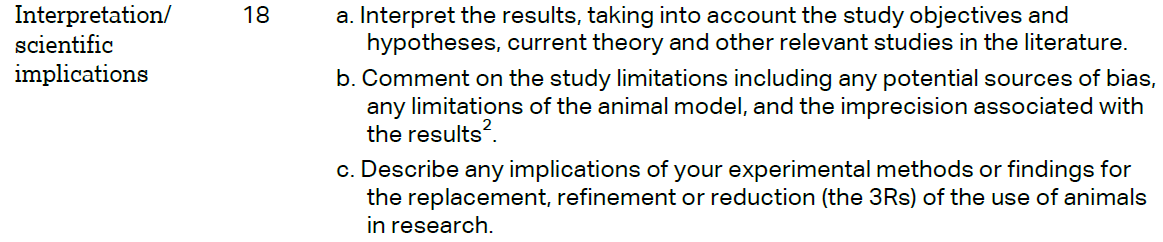 | | | Pages 16-20  Discussion | |
| 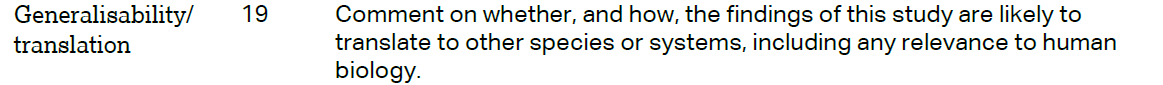 | | | Page 20  Conclusion | |
| 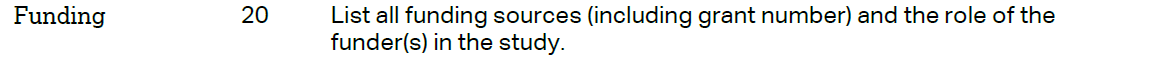 | | | Pages 20 and 21  Financial Support |  |


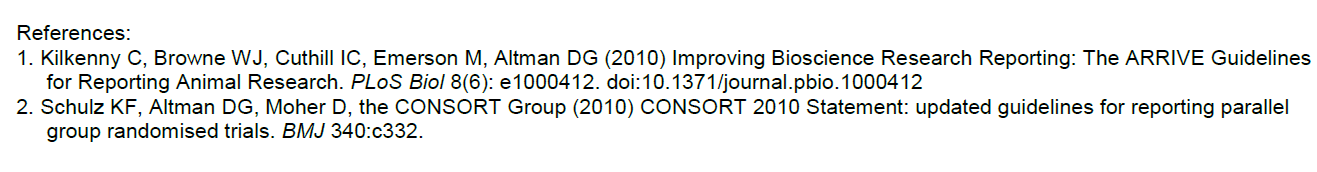

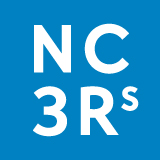

Supplement: S1 ARRIVE Checklist — (DOC) [file pone.0132038.s001.doc]
